# Impact of the COVID-19 pandemic on the incidence, etiology, and antimicrobial resistance of healthcare-associated infections in a critical care unit in Western Qatar

**DOI:** 10.5339/qmj.2023.2

**Published:** 2022-12-23

**Authors:** Humberto Guanche Garcell, Jameela Al-Ajmi, Ariadna Villanueva Arias, Joji C Abraham, Angel M Felipe Garmendia, Tania M Fernandez Hernandez

**Affiliations:** ^1^Infection Control Department, The Cuban Hospital. E-mail: humbertoguanchegarcell@yahoo.es ORCID: https://orcid.org/0000-0001-7279-0062; ^2^Corporate Infection Control Department, Hamad Medical Corporation, Qatar.

**Keywords:** Healthcare-associated infections, Device-associated infection, COVID-19, etiology, antimicrobial resistance, intensive care unit, incidence, Qatar

## Abstract

Background: Healthcare-associated infections (HAIs) in critical patients affect the quality and safety of patient care and increase patient morbidity and mortality. During the COVID-19 pandemic, an increase in the incidence of HAIs, particularly device-associated infections (DAIs), was reported worldwide. In this study, we aimed to estimate the incidence of HAIs in an intensive care unit (ICU) during a 10-year period and compare HAI incidence during the preCOVID-19 and COVID-19 periods.Methods: A retrospective, observational study of HAIs in the medical–surgical ICU at The Cuban Hospital was conducted. DAIs included central line-associated bloodstream infections (CLABSI), catheter-associated urinary tract infections (CAUTI), and ventilator-associated pneumonia (VAP). Data included the annual incidence of HAIs, etiology, and antimicrobial resistance, using definitions provided by the Centers for Disease Control and Prevention, except for other respiratory tract infections (RTIs).Results: 155 patients with HAI infections were reported, from which 130 (85.5%) were identified during the COVID-19 period. The frequencies of DAIs and non-DAIs were higher during the COVID-19 period, except for *Clostridium difficile* infections. Species under *Enterobacter*, *Klebsiella*, and *Pseudomonas* dominated in both periods, and higher frequencies of *Acinetobacter*, *Enterococcus*, *Candida*, *Escherichia coli*, *Serratia marcescens*, and *Stenotrophoma maltophila* were noted during COVID-19 period. Device utilization ratio increased to 10.7% for central lines and 12.9% for ventilators, while a reduction of 15% in urinary catheter utilization ratio was observed. DAI incidence was higher during the COVID-19 pandemic, with risks for CLABSI, VAP, and CAUTI increased by 2.79 (95% confidence interval, 0.93–11.21; *p* < 0.0050), 15.31 (2.53–625.48), and 3.25 (0.68–31.08), respectively.Conclusion: The incidence of DAIs increased during the pandemic period, with limited evidence of antimicrobial resistance observed. The infection control program should evaluate strategies to minimize the impact of the pandemic on HAIs.

## Introduction

Healthcare-associated infections (HAIs) in critical patients impact the quality and safety of patient care by increasing the risk of morbidity and mortality. Moreover, HAI decreases efficiency in the healthcare system due to prolonged hospital stays, antimicrobial use, and other cost-related issues.^1^

Reducing device-associated infections (DAIs) has become a priority for infection prevention in critical patients. The incidence of central line-associated bloodstream infection (CLABSI), catheter-associated urinary tract infection (CAUTI), and ventilator-associated pneumonia (VAP) constitute key infection control indicators mandated by the Joint Commission International.^2^ Before the pandemic, DAI prevention and control showed progress in reducing the incidence of DAIs and other adverse outcomes, according to various reports.^3–6^

The COVID-19 pandemic has significantly disrupted global healthcare systems. Infection prevention and control programs have been seriously affected, with increased HAI incidence and compromised staff safety and healthcare efficiency.^6–14^ A recent publication by the Center for Disease Control (CDC/NHSN) reported a higher incidence of DAIs in 2020 than in 2019, emphasizing ventilator-associated events (VAEs) and CLABSI.^3^ Moreover, studies published on HAIs in various settings during the pandemic describe similar findings, suggesting a convergence of factors that generate a high-risk environment for acquiring infections.^6–16^

The Cuban Hospital (Dukham, Qatar) was dedicated to providing care to COVID-19 patients from 2020 to 2021. Originally a 75-bed public hospital, it expanded to 385 beds, including tents, after becoming part of the Hamad Medical Corporation, the main healthcare provider in Qatar and a Joint Commission International-accredited organization.^17^ The facility infection control program is guided by the corporate infection control program. Data from infection surveillance suggest an increase in the incidence of HAIs in the pandemic compared to the prepandemic period, mostly in critically ill patients.

Considering the evidence, we conducted a study aiming at describing the incidence of healthcare-associated infections in the ICU during a 10-year follow-up period and to compare the HAI incidence during the preCOVID-19 and COVID-19 periods.

## Methods

This is a retrospective, observational study of HAIs in the medical–surgical intensive care unit (ICU) at The Cuban Hospital. During the preCOVID-19 period (2012–2019), the ICU had a 6-bed capacity, which was expanded to 85 beds during the COVID-19 pandemic (2020–2021).

Data was collected from the Infection Control Department records. The surveillance system was developed and set up by an infection control practitioner and a hospital epidemiologist. Due to potential variations in the infection control staff from 2017 to 2019, data were validated by reviewing patient medical records and reports from the microbiologic laboratory. Data collected included the annual incidence of HAIs, etiology, and antimicrobial resistance. Data on etiology and antimicrobial resistance were verified using the patient electronic medical record. HAI was confirmed using the Centers for Disease Control and Prevention (USA) definitions adopted by the corporate infection control program, except for other respiratory tract infections (RTIs).^18^ RTIs were confirmed when a positive culture from the respiratory tract (sputum, tracheal aspirate, or other respiratory samples) showed evidence of sepsis (clinical symptoms or sepsis laboratory markers), and VAEs were ruled out. Device-associated infections, such as CLABSI, CAUTI, VAP, and non-DAIs, such as skin infections, decubitus ulcer infections, and symptomatic urinary tract infections, were reported.

### Analysis

Descriptive statistical methods were used. Data on DAIs were presented as rates (1000 procedures days) and percentile distribution, while non-DAIs as a percentage. Statistical analyses were performed using the statistical package SPSS, version 22 (SPSS Inc., Chicago, Illinois). Relative risk (RR) ratios, 95% confidence intervals (CIs), and *P*-values were determined. We used the two-tailed Student's *t*-test to compare differences between HAI incidence during the preCOVID-19 and COVID-19 periods. Two-tailed *P-*values less than 0.05, the significance level (α), was considered statistically significant.

## Results

During the study period, 155 patients with HAIs were reported, of which 130 (85.5%) were identified during the COVID-19 pandemic period. The frequencies of DAI and non-DAI infections were higher during the COVID-19 pandemic, except for *Clostridium difficile* infection. The most frequently reported infection was other RTIs, which were not classified as VAP. In patients with coronavirus infection, there were two confirmed cases of bloodstream infection (BSI) related to peripheral venous lines, four cases with decubitus ulcer infection, five cases of skin infection associated with tracheostomy, and one case of symptomatic urinary tract infection (Table 1).

The most frequent etiological agents in both periods were species under *Enterobacter*, *Klebsiella*, and *Pseudomonas*. Higher frequencies of *Acinetobacter*, *Enterococcus*, *Candida*, *Escherichia coli*, *Serratia marcences*, and *Stenotrophoma maltophila* were observed during the COVID-19 pandemic (Table 2).

Table 3 describes the DAI incidence and utilization ratio during the 10-year study period. The incidence was 0.8, 2.6, and 2.9 per 1000 urinary catheters, central lines, and ventilators for CAUTI, CLABSI, and VAP, respectively. The device utilization ratio was 0.49 for urinary catheters, 0.32 for central lines, and 0.31 for ventilators.

In the preCOVID-19 (2012–2019) and COVID-19 (2020–2021) periods, 2255 and 4048 central line days, 2124 and 3888 ventilator days, and 3708 and 5106 urinary catheter days, respectively, were reported. The utilization ratio increased by 10.7% for central lines and 12.9% for ventilators, while a reduction of 15% in urinary catheter utilization ratio was observed. DAI incidence was higher during the COVID-19 pandemic, with a significant increase for VAP [from 0.47 to 7.2 per 1000 ventilator days], and CLABSI [from 1.77 to 4.94 per 1000 central line days], and CAUTI [from 0.54 to 1.76 per 1000 urinary catheter days]. These represent an increase in the risk of infection by 2.79 (95% CI, 0.93–11.21; *p* < 0.05), 15.31 (2.53–625.48; *p* < 0.00), and 3.25 (0.68–31.08; *p* < 0.012) for CLABSI, VAP, and CAUTI respectively (*p* < 0.00) (Table 4).

Table 5 shows DAI rates from previously published reports.^6,10,18–21^

## Discussion

This is the first report that provides data useful in benchmarking the incidence of HAIs and the impact of coronavirus infection in a single facility in Qatar. It highlights the increased risk of infection related to the COVID-19 pandemic, particularly concerning incidence of VAP and CLABSI.

The incidence of DAIs during the study period shows lower results than those reported by the International Nosocomial Infection Control Consortium (2013–2018) and the Kingdom of Saudi Arabia (2013–2016).^19,22^ The incidence of CLABSI and CAUTI were lower than that reported by National Healthcare Safety Network (NHSN, USA) (2012), while for VAP it was higher than that reported by NHSN (2013).^20,21^ The pandemic-related factors that contributed to the increase in the pooled 10-year mean outcomes should be considered for setting the facility's infection prevention program goals.

The ICU changed in complexity substantially during the pandemic compared to the prepandemic period. This is mainly related to patients’ population characteristics, from a prepandemic population with low HAI risk to COVID-19 patients with high HAI risk. This risk is determined by the COVID-19 clinical severity, treatment with antibiotics and corticosteroids, and the need for invasive devices, especially mechanical ventilation and central venous and arterial lines. These factors play critical roles in increasing HAI incidence during the pandemic, in addition to non-compliance with infection control practices.^3,23,24^

It is worth noting the frequency we observed of other respiratory infections, not classified as VAP, which may correspond to tracheobronchitis in ventilated patients.^25,26^ A multicenter study conducted in 36 European ICUs showed an increased incidence of ventilator-associated lower RTIs in COVID-19 patients and 28% of these infections were related to tracheobronchitis.^25^

Although few cases have been reported in the COVID-19 period, the incidence of confirmed bloodstream infections related to peripheral lines should be highlighted. Previous references to the risk of infection associated with these devices require prevention practices like those recommended for central lines.^27,28^

The increase in CLABSI and VAP incidence and the non-increase of CAUTI incidence during the COVID-19 pandemic have been reported previously.^3,12^ Concerning the risk of CAUTI, it is worth considering that although urinary catheters were intensively used in patients with coronavirus, the utilization ratio was lower than that observed during the prepandemic period.

The etiologies of the reported infections were similar to previously described agents, except for the high frequency of infections caused by *Candida* species, especially cases of CLABSI caused by *Candida auris*. Most of the patients with *Candida auris* infection had previously reported colonization.^29–31^

An increase in antimicrobial resistance has been reported as a principal consequence of the pandemic,^29,31^ including in bacterial and fungal infections. However, the number of isolated drug-resistant organisms during the study cannot be considered conclusive data to confirm this finding. However, the increase in the frequency of multidrug-resistant species like *Enterobacter*, *Klebsiella*, *Pseudomonas*, *Serratia marcescens*, and *Stenotrophoma maltophila* should point to the need to strengthen our policies and practices to contain the emergence of antimicrobial resistance.

The study has a few limitations, including a retrospective observational design and single-center data collection, which limits data comparison to ICUs with similar characteristics. The analysis should also consider the few multidrug-resistant isolated organisms. The impact of data collection by various infection control staff (mainly for 2017–2019) was minimized by accurate data validation. The main strength of the study is the use of a standardized surveillance system and procedures guided by a corporate infection control program

## Conclusion

The incidence of DAIs increased during the pandemic period, compared to the prepandemic period. Limited evidence of the impact of the pandemic on antimicrobial resistance was observed. The infection control program should evaluate strategies to minimize the impact of pandemics on HAIs.

### Acknowledgment

To Prof. Alexis Gonzalez Velázquez for the proofreading of the manuscript.

## Figures and Tables

**Table 1:** Healthcare-associated infections in the preCOVID-19 (2012–2019) and COVID-19 (2020–2021) periods at The Cuban Hospital Medical–Surgical Intensive Care Unit

Infections	PreCOVID-19n = 22	COVID-19n = 130

DAI		

CAUTI	2 (9.1%)	13 (10.0%)

CLABSI	1 (4.5%)	30 (23.1%)

VAP	1 (4.5%)	30 (23.1%)

Blood stream infection related to peripheral venous line	-	2 (1.5%)

Other healthcare-associated infections		

Others respiratory infections	17 (77.3%)	46 (35.4%)

*Clostridium difficile* infections	1 (4.5%)	-

Decubitus ulcer infection	-	4 (3.1%)

Skin infections	-	5 (3.8%)

Symptomatic urinary tract infection 1b	-	1 (0.8%)


CAUTI - Catheter-associated urinary tract infection, CLABSI - Central line-associated bloodstream infection, VAP - Ventilator-associated pneumonia, DAI - Device- associated infection

**Table 2:** Etiology of healthcare-associated infections in the preCOVID-19 (2012–2019) and COVID-19 (2020–2021) periods at The Cuban Hospital Medical–Surgical Intensive Care Unit

	PreCOVID-19		COVID-19	

isolate	Total No. isolates	MDRO (%)*	Total No. isolates	MDRO (%)*

Bacterial				

*Acinetobacter* spp.	0		6	0

*Citrobacter* spp.	0		2	0

*Enterobacter* spp.	3	33.3	12	91.7

*Enterococcus* spp.	0		9	11.1

*Escherichia coli*	0		6	83.3

*Klebsiella* spp.	6	16.7	30	46.7

*Morganella morgani*	1	100	1	100

*Proteus* spp.	2	50	0	

*Pseudomonas* spp.	5	20	42	40.5

*Serratia marcescens*	2	50	11	100

*Staphylococcus aureus*	2	50	3	66.6

*Stenotrophoma maltophila*	1	100	9	66.7

*Clostridium difficile*	1	100	0	0

Fungal**				

*Candida* spp.	0		13	

*Trichosporon asahii*	1		0	


* Proportion of isolates with multidrug-resistance, ** resistance to antifungals not presented, #spp. refers to species, MDRO - Multidrug-resistant organisms

**Table 3:** Pooled means and key percentiles of the distribution of DAI rates and device utilization ratios at The Cuban Hospital Medical–Surgical Intensive Care Unit 2012–2021

Infection	Infections	Device days	Pooled mean	10%	25%	50% (median)	75%	90%

CAUTI	15	8,814	0.8	0.0	0.0	0.0	2.2	2.6

CLABSI	31	6,303	2.6	0.0	0.0	0.0	7.1	8.4

VAP	31	6,012	2.9	0.0	0.0	0.0	6.3	13.5

Devices	Patients days	Devicedays	Pooled mean	10%	25%	50% (median)	75%	90%

Urinary catheter	19,204	8,814	0.49	0.37	0.42	0.46	0.55	0.66

Central line	19,204	6,303	0.32	0.25	0.30	0.33	0.34	0.35

Ventilator	19,204	6,012	0.31	0.20	0.21	0.30	0.39	0.49


CAUTI - Catheter-associated urinary tract infection, CLABSI - Central line-associated bloodstream infection, VAP - Ventilator-associated pneumonia, DAI - Device-associated infection

**Table 4:** Device utilization ratio and infection rate (per 1000 device days) during 2012–2019 (preCOVID period) and 2020–2021 (COVID-19 period)

	PreCOVID-19 (2012-2019)			COVID-19 (2020-2021)			RR (95% CI)

DAI Type	Device days, n	Infections, n	HAI rate	Device days, n	Infections, n	HAI rate	

CAUTI	3708	2	0.54	5106	9	1.76	3.25 (0.68–31.08)***

CLABSI	2255	4	1.77	4048	20	4.94	2.79 (0.93–11.21)*

VAP	2124	1	0.47	3888	28	7.20	15.31 (2.53–625.48)**


* *p* < 0.05 ** *p* = 0.00 *** *p* = 0.12, CAUTI - Catheter-associated urinary tract infection, CLABSI - Central line-associated bloodstream infection, VAP - Ventilator-associated pneumonia, DAI - Device-associated infection, RR - Risk ratio, CI - Confidence interval

**Table 5:** Summary data of device utilization ratio and infection rate (per 1000 device days) in various reports

Reports^*^	Population	CLABSI		VAP		CAUTI	

		Infection Rate	Utilization ratio	Infection Rate	Utilization ratio	Infection Rate	Utilization ratio

NHSN 2012	Medical-surgical ICU ≤ 15 beds			1.1	0.24		

	Medical-surgical ICU>15 beds			0.9	0.34		

NHSN 2013	Medical-surgical ICU ≤ 15 beds	0.8	0.37			1.3	0.54

	Medical-surgical ICU>15 beds	0.8	0.49			1.7	0.63

INICC 2013-2018	Medical surgical ICUs	4.4	0.53	11.13	0.38	2.97	0.67

KSA 2013-2016	Medical-surgical ICU ≤ 15 beds	0-6.9	0.58	18.1-26.6	0.54	2.3-7.19	0.75

	Medical-surgical ICU>15 beds	0-22.8	0.33-0.68	0.9-186	0.05-0.94	0-11.75	0.06-0.85

US hospitals 2019-2020	Critical care units 78 US hospital PreCovid-19 period	0.68	0.43			0.88	0.48

	Critical care units 78 US hospital Covid-19 period	1.16	0.48			0.90	0.51

INICC 2022	Medical-surgical ICUs PreCovid-19 period	2.54		9.71		1.64	

	Medical-surgical ICUs COVID-19	4.73		12.58		1.43	


* References 6, 10, 16–19 CAUTI - Catheter-associated urinary tract infection, CLABSI - Central line-associated bloodstream infection, VAP - Ventilator-associated pneumonia, NHSN- National Healthcare safety Network (Center for Disease Control, USA), INICC - Internation Infection Control Consortium (Argentina), KSA - Kingdom of Saudi Arabia, ICU - Intensive care Unit
